# On the Efficacy of Cold Astringent Injections into the Cavity of the Uterus in Arresting Uterine Hæmorrhage

**Published:** 1846-04

**Authors:** L. G. Ray

**Affiliations:** Paris, Ky.


14. *On the efficacy of Cold Astringent Injections into the cavity of the Uterus in arresting Uterine Hæmorrhage.* By L. G. Ray, M. D., of Paris, Ky.—At the commencement of our practice, having failed, in some cases, to arrest this fearful malady, we were induced to resort to a remedy, which, though novel in its application, seemed peculiarly fitted to meet the indications of cure, by arresting *instantly* the flow of blood. It is simple, easily applied, and *most efficacious* in its results, and hence should command the attention of the profession. We cannot better illustrate its effects than by giving a case.

Mrs. F——, æt. 43, had menstruated regularly when not pregnant, missed two periods, and was inclined to the opinion that she was again pregnant. Was attacked with uterine hæmorrhage, which soon became alarming. We saw her six hours after her attack; found her with feeble, frequent (uncountable) pulse, anxious countenance, hurried respiration, extremities and whole surface cold, covered with a dripping and cold perspiration, partial syncope taking place at every movement she made, without pain, os tincæ soft and relaxed as also the body, hæmorrhage still progressing. In this state of things we used, instanter, a saturated solution of sub. acet. plumb. in acetic acid, cold; which we threw into the cavity of uterus by a curved pipe womb syringe; pain of a sharp character instantly ensued, followed by a sudden contraction of uterus, and *entire arrest* of the alarming hæmorrhage. Brandy, with a little tr. opi., internally, frictions to surface, and sinapism, to extremities, restored heat and comfort, in a short time to the almost dying lady. Six hours after use of injection, found her comfortable, cheerful, pulse a little more frequent than natural. No return of hæmorrhage.* Ordered an occasional aperient, and rest. Mur. tr. ferri, in doses of 30 drops thrice daily; a little brandy at meals, with generous diet. This lady recovered her usual health and vigor in a few weeks without any bad effects from the use of injection.

This practice we have followed for the last fifteen years, in alarming cases of uterine hæmorrhage, without any unpleasant consequences following, and with the *most complete and perfect success.* We have used it in hæmorrhages following and accompanying abortion, and from retained placenta, with equal success,—and we have no doubt it may be safely and suc-

* In this case, it must be borne in mind that brandy, laudanum, and various other remedies had been used before we arrived, without one particle of relief.

cessfully used in all cases of uterine hæmorrhage accompanied with a relaxed and atonic state of the organ.

Since writing out this case, we have used the remedy in a case of engorged uterus with some tenderness, accompanied with uncontrollable hæmorrhage; and although it succeeded in arresting the hæmorrhage, it caused our patient most intense pain, which was only relieved by two tea-spoonsful of acet. tr. opium, with a ½ grain suph. morphia. After this, she had a speedy recovery.

